# Celebrating a quarter century of the Injury Free Coalition for Kids®

**DOI:** 10.1186/s40621-020-00248-z

**Published:** 2020-06-12

**Authors:** Lois K. Lee

**Affiliations:** 1 Division of Emergency Medicine, Boston Children’s Hospital, Boston, MA USA; 2 Departments of Pediatrics and Emergency Medicine, Harvard Medical School, Boston, MA USA

“We choose to go to the moon in this decade and do the other things, not because they are easy, but because they are hard, because that goal will service to organize and measure the best of our energies and skills, because that challenge is one that we are willing to accept, one we are unwilling to postpone, and one which we intend to win.” -- John F. Kennedy.

This year we celebrate the 25th anniversary of the Injury Free Coalition for Kids®. Although we are commemorating this anniversary in 2020, the Injury Free story really began in 1981 when Dr. Barbara Barlow, founder of the Injury Free Coalition for Kids®, established the Injury Prevention Program at Harlem Hospital in New York City, NY. This Program instituted a population-based pediatric injury surveillance system in Harlem to identify the leading mechanisms of injury for children and youth in this area to inform and evaluate injury prevention efforts (Durkin et al., 1994). Based on this initial work, in 1988 the Robert Wood Johnson Foundation (RWJF) provided a 3-year grant to further establish and develop the Harlem Hospital Injury Prevention Program (HHIPP) (Pressley et al., 2005). Initiatives implemented by the HHIPP during this time included window guard campaigns; the Kids, Injuries and Street Smarts (KISS) Program; Burn Prevention Curriculum and Smoke Detector Distribution; Harlem Alternative to Violence Program; Critical Incident Stress Management Teams; youth sports programs; and the Greening of Harlem Program. In addition to these programs, Dr. Barlow and the HHIPP innovatively worked to eliminate unsafe community spaces and replace them with safe spaces for children to play by establishing community coalitions to build playgrounds and improve the community environment (Laraque et al., 1995).

Based on the successful work of the HHIPP, the RWJF funded a 3-year “replication program” in 1995 to expand this injury prevention program model to 5 other sites in Atlanta, GA, Chicago, IL, Kansas City, MO, Los Angeles, CA, and Pittsburgh, PA. None of these cities had population-based injury surveillance systems or active injury prevention programs at that time. In 1997, this multi-site injury prevention initiative was given the name the “Injury Free Coalition for Kids®.” Then in 2001 RWJF funded the National Program Office and “Dissemination of a Model Injury Prevention Program,” to expand Injury Free from 15 to 40 sites by 2003. Now the Injury Free Coalition for Kids® has sites at over 40 institutions in the U.S. and Canada, all working with their communities to decrease injuries to children and youth. (Pressley et al., 2005)

We have included two seminal articles related to the establishment of Injury Free in this edition of *Injury Epidemiology (Durkin et al.,*^1994^*;Laraque et al.,*^1995^*)*.

These programs were developed on the foundations of Injury Free’s ABC’s of injury prevention model:

- **A, “analyze the data”** on injuries in the community
- **B, “build a coalition”** by engaging the local community to understand and respond to the problems
- **C, “communicate the problem”** to educate political leaders and the community about pediatric injuries
- **D,** “**develop the interventions”** to decreased injuries to community children
- **E, “evaluate the programmatic efforts”** to evaluate the impact of the interventions (Pressley et al., 2005).

These principles continue to guide the Injury Free sites as they work to prevent injuries in their communities and nationally.

As we celebrate the 25th anniversary of the Injury Free Coalition for Kids, we also want to remember Dr. Joseph J. Tepas, III, who died on December 20, 2019. Dr. Tepas was a pediatric surgeon and a leader in the field of pediatric trauma. He was instrumental in founding the first pediatric-specific national trauma registry, the National Pediatric Trauma Registry. He served as the supplement editor for the annual Injury Free meeting proceedings published in the *Journal of Trauma and Acute Care Surgery* from 2007 to 2017. Publication of this meeting supplement, now in *Injury Epidemiology,* continues to be an important venue for demonstrating and disseminating our injury prevention work and research. We honor his unmeasurable contributions to improving the lives of children and are grateful for all his service as a member and editor for the Injury Free Coalition for Kids®.

Visionaries like Dr. Barlow and Dr. Tepas have made a difference in the lives of innumerable children and families as they have forged new frontiers in pediatric trauma care and injury prevention. They did not choose this path because it was easy, but because it was hard, and because they it was one they were unwilling to postpone. After 25 years their work has accomplished a great deal, but there is still much to be done. And the Injury Free Coalition for Kids® continues to accept this challenge, knowing it is not easy, but that it is hard. Because our goal is not the “moon,” but for our children to leave in a world that is Injury Free.

## Data Availability

Not applicable.
