# Correction to: Celebrating a quarter century of the Injury Free Coalition for Kids®

**DOI:** 10.1186/s40621-020-00285-8

**Published:** 2020-10-12

**Authors:** Lois K. Lee

**Affiliations:** 1 Division of Emergency Medicine, Boston Children’s Hospital, Boston, MA USA; 2 Departments of Pediatrics and Emergency Medicine, Harvard Medical School, Boston, MA USA

**Correction to: Inj Epidemiol 7, 29 (2020)**

**https://doi.org/10.1186/s40621-020-00248-z**

After publication of this supplement article (Lee 2020), it was brought to our attention that there is an error in the last sentence.

The sentence currently reads:

Because our goal is not the “moon,” but for our children to leave in a world that is Injury Free.

The sentence should read:

Because our goal is not the “moon,” but for our children to live in a world that is Injury Free.

This has now been included in this correction article.
